# Lipid-modified G4-decoy oligonucleotide anchored to nanoparticles: delivery and bioactivity in pancreatic cancer cells

**DOI:** 10.1038/srep38468

**Published:** 2016-12-08

**Authors:** S. Cogoi, U. Jakobsen, E. B. Pedersen, S. Vogel, L. E. Xodo

**Affiliations:** 1 Department of Medical and Biological Sciences, P. le Kolbe 4, 33100 Udine, Italy; 2 Nucleic Acid Center, Institute of Physics and Chemistry, University of Southern Denmark, DK-5230 Odense M, Denmark; 3 PET & Cyclotron Unit, Department of Nuclear Medicine, Odense University Hospital, Sdr. Boulevard 29, 5000 Odense C, Denmark

## Abstract

*KRAS* is mutated in >90% of pancreatic ductal adenocarcinomas. As its inactivation leads to tumour regression, mutant *KRAS* is considered an attractive target for anticancer drugs. In this study we report a new delivery strategy for a G4-decoy oligonucleotide that sequesters MAZ, a transcription factor essential for *KRAS* transcription. It is based on the use of palmitoyl-oleyl-phosphatidylcholine (POPC) liposomes functionalized with lipid-modified G4-decoy oligonucleotides and a lipid-modified cell penetrating TAT peptide. The potency of the strategy in pancreatic cancer cells is demonstrated by cell cytometry, confocal microscopy, clonogenic and qRT-PCR assays.

Pancreatic ductal adenocarcinoma (PDAC) is a human malignancy with a very poor prognosis and an average survival after diagnosis of less than 6 months^1^. As current treatments are not very effective, surgery is the main option^2^, so there is an urgent need to develop new therapies. The main genetic lesion present in >90% of PDAC patients is a mutation in the *KRAS* proto-oncogene, in exon 1, primarily at codon 12 or, at lower frequency, at codons 13 or 61^3^. Several studies have demonstrated that mutant *KRAS* is a major driver of PDAC^3^,^4^,^5^ and that the expression of mutant *KRAS*^G12D^ in the pancreas of transgenic mice causes intraepithelial neoplasia lesions that progress into full malignancy^5^. The expression of *KRAS*^G12D^ is required for tumour maintenance, and the extinction of the gene leads to a rapid tumour regression^5^. These data suggest that pancreatic cancer cells are “addicted” to mutant *KRAS*^G12D^. Indeed, recently De Pinho and co-workers reported that *KRAS*^G12D^ reprograms the metabolism of pancreatic cancer cells, in order to meet their increased anabolic need to fuel an enhanced rate of growth^6^. They found that *KRAS*^G12D^ increases (i) the glucose transporter *Glut*-1 and the glycolytic flux; (ii) the synthesis of ribose 5-phosphate and purine and pyrimidine nucleobases, and (iii) the consumption of glutamine to maintain the redox balance^6^,^7^. Given the strong impact of *KRAS*^G12D^ on the metabolism of PDAC cells, this oncogene is an attractive target for new anticancer drugs. For a rationale design of anticancer drugs we focused on the *KRAS* promoter, as it contains three G4 motifs, of which the one most close to TSS, G4-proximal, has been extensively studied^8^,^9^,^10^. G4-proximal is located between −144 and −117 upstream of TSS, overlaps a nuclease hypersensitive element (NHE) and is recognized by several nuclear proteins including MAZ, PARP-1, Ku70/Ku80 and hnRNP A1^10^. G4 proximal is composed of six runs of guanines (G-runs 1 to 6) and can fold into different G-quadruplex structures, as suggested by primer extension experiments^11^ (Supplementary Information, Fig. S1). DMS footprinting and CD experiments showed that sequence G4-proximal folds into a parallel 1/1/11 G-quadruplex with a kinked thymine in one strand, two 1-nt and one 11-nt loops (formed by G-runs 1-2-3-5, Q_1_, *T*_M_ = 75 °C in 100 mM KCl, Fig. S1)^9^. Further analyses showed that G4-proximal exhibits a remarkable structural polymorphism, as two truncated G4-proximal sequences composed by G-runs 1-2-3-4 and 2-3-4-5 fold also into a G-quadruplex, respectively with a parallel, *T*_M_ = 56 °C, 100 mM KCl (Q_2_) and mixed parallel/antiparallel, *T*_M_ = 50 °C, 100 mM KCl (Q_3_) topologies (Fig. S1)^11^,^12^. Of the three G-quadruplexes associated to G4-proximal, the one formed by G-runs 2-3-4-5 showed the strongest affinity for MAZ: a transcription factor that binds to G4-proximal and activates the transcription of *KRAS*^12^. This truncated sequence of G-proximal exists in equilibrium between uni- and bi-molecular G-quadruplex^12^. The bimolecular quadruplex has been recently resolved by NMR^13^. We also demonstrated that the insertion of two *para*-TINA (twisted intercalated nucleic acid) units in the sequence, shifts the equilibrium towards the unimolecular structure (*T*_M_ = 79 °C, 100 mM KCl)^12^. The TINA-modified quadruplex called **2998** showed a strong bioactivity, as it suppressed *KRAS*, inhibited proliferation and activated apoptosis in Panc-1 cancer cells^12^. Furthermore, injected into a Panc-1 xenograft, **2998** inhibited tumour growth and increased the survival time of the mice by ~50%^12^. This modified oligonucleotide is expected to act through a decoy mechanism by sequestering MAZ and thus depriving the promoter of an essential transcription factor^14^. To improve the delivery of **2998**, which in previous studies was given to the cells complexed to jet-PEI (PolyPlus), we designed a new strategy based on the use of palmitoyl-oleyl-phosphatidylcholine (POPC) liposomes to which we anchored noncovalently the oligonucleotide modified with a lipid moiety. Here, we demonstrate that the G4-decoy **2998** anchored to the liposomes efficiently internalize in pancreatic Panc-1 cancer cells where it reduces the metabolic activity, the clonogenicity as well as the level of *KRAS* transcript.

## Results and Discussion

We have previously demonstrated that the G4-decoy oligonucleotide **2998** (Fig. S1) delivered with polyethylenimine (jet-PEI) activates through a decoy mechanism a strong apoptotic response in Panc-1 cells and reduces the growth of a Panc-1 xenograft in mice^12^. To improve the delivery of the G4-decoy, we have designed a transport system based on the low toxicity of palmitoyl-oleyl-phosphatidylcholine (POPC) liposomes in combination with surface attached functionalities^15^. POPC liposomes are functionalized with a cell-penetrating peptide (CPP), either the trans-activator of transcription of the human immune-deficiency virus (TAT) or the cationic octaarginine peptide (R8), and G4-decoy oligonucleotide **2998**^16^,^17^,^18^,^19^,^20^.

**Figure 1:**
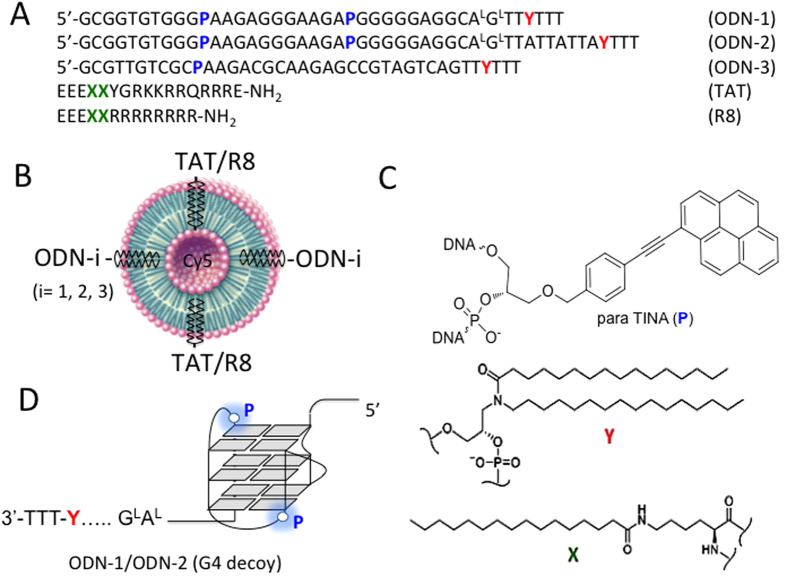
(**A**) Oligonucleotides and peptides used to functionalize POPC liposomes. ODN-1 and ODN-2 have the sequence of *KRAS* G4-proximal motif, the portion composed by the G-runs 2-3-4-5^8^,^12^. The oligonucleotides are chemically modified as they contain 2 *para*-TINA insertions (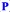), two locked nucleic acid modifications (A^L^G^L^) and one 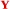 insertion with two saturated palmityl chains in order to anchor the oligonucleotides to the liposomes. ODN-3 is a non-G4 oligonucleotide used as control. The sequence of the two CPP peptides (TAT and R8) is shown. They contain two lipid insertions 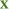 in order to anchor them to the liposomes; (**B**) POPC liposomes spontaneously self-assembles into spherically closed bilayer membrane on the surface of which CPP and G4-decoy ODNs are attached through their lipid modifications (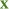 or 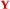); (**C**) Structure of para TINA (P) inserted in the G4-decoy oligonucleotides; structures of lipid insertion 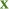 contained in R8 and TAT and 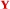 contained in ODN1-3; (**D**) Proposed structure for the G4-decoys ODN-1 and ODN-2.

As the synthesis of bioconjugates between the G4-oligonucleotide and CPP is very demanding and would require a new synthesis for each new peptide or oligonucleotide used in the bioconjugate, a delivery strategy based on POPC liposomes is an attractive alternative^21^,^22^,^23^. To functionalize the liposomes a non-covalent membrane anchoring strategy for both the G4-oligonucleotide and the CPP was employed. Both functionalities, peptide and oligonucleotide, were chemically modified with a palmityl membrane anchor to allow their rapid attachment to the liposome surface^21^,^22^,^23^. The strategy is illustrated in Fig. 1. POPC liposomes are treated with the lipid-modified G4-oligonucleotide and peptide that spontaneously anchor to the liposome surface^24^. As the G4-decoys are not covalently attached to the liposomes, they can move freely on the lipid surface and interact efficiently with the target proteins. The membrane anchor of the G4-decoy consists of a 3-amino-1,2-propanediol unit with two saturated palmityl chains (membrane anchor **Y**)^25^. We prepared three palmityl-modified oligonucleotides (Table 1). ODN-1 and ODN-2 were designed with: (i) the sequence of truncated G4-proximal comprising G-runs 2-3-4-5^12^; (ii) two *para*-TINA (**P**) units to stabilize the unimolecular folding of the oligonucleotide^12^; (iii) a membrane anchor **Y** followed by three thymidines at the 3′ end. As previously found, the additional TTT nucleotides prevent possible oligonucleotide self-aggregation and reduce the detergent properties of the lipid-modified G4-oligonucleotide^21^,^22^,^23^. Between **Y** and the G4-oligonucleotide a spacer of either four (A^L^G^L^TT, ODN-1) or eleven (A^L^G^L^TTATTATTA, ODN-2) nucleotides was introduced. Two nucleotides of the spacer have been replaced with locked-nucleic acids (LNA) analogues (AG→A^L^G^L^), to avoid possible nuclease cleavage of the G4-motif from the lipid moiety^26^,^27^,^28^. The effect of LNA modifications on the susceptibility to nuclease degradation of DNA in duplex and quadruplex conformations has been previously reported^12^,^13^,^14^,^15^,^16^,^17^,^18^,^19^,^20^,^21^,^22^,^23^,^24^,^25^,^26^,^27^,^28^. The introduction of one or two terminal LNAs in the *NF-kB* duplex decoy was sufficient to markedly increase the oligonucleotides stability against exo- and endo-nucleases^27^,^28^. Similarly two LNAs at the 3′ end of **2998** showed a high stability in serum^12^. In the light of these data we designed our lipid-modified decoys with two LNAs placed outside the G4-motif to avoid a possible effect of the sugar modification on the quadruplex structures^29^,^30^. As for ODN-3, it bears the same modifications as ODN-1 and ODN-2 but was designed with a random sequence that does not allow any folding (this oligonucleotide was used as a control). A similar strategy was used for the lipid modification of TAT- or R8-derived peptide. The two palmityl-modifications (**X**) were incorporated close to the *C*-terminus, followed by three additional glutamic acid residues to avoid self-aggregation of the peptide. At the same time, the three E residues provide additional negative charges and reduce the peptide’s net positive charge from 7+ to 4+ thus decreasing the toxicity usually associated with strongly positive charged peptides (Fig. 1). The molecular weight of the synthesized lipid-modified oligonucleotides and peptides, after purification, was checked by MALDI-TOF mass spectrometry. The difference between theoretical and experimental masses was in general <0.01% (Table 1).

**Figure 2:**
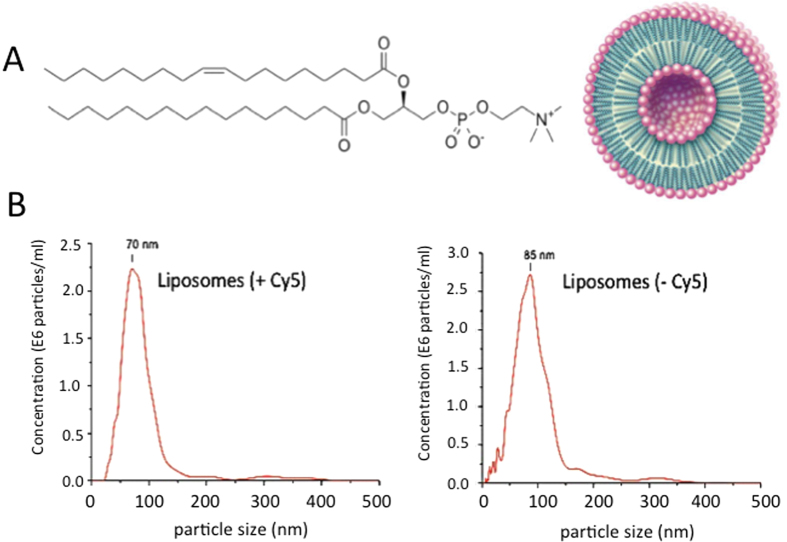
(**A**) Structure of palmityl oleyl-phosphatidylcholine (POPC) and representation of the liposome; (**B**) Nanoparticle Tracking Analysis shows that the POPC liposomes with 0.5 mol % Cy5 form 70 nm nanoparticles whereas liposomes without Cy5 form 85 nm nanoparticles. Cy5 is cyanine 5, its structure is reported in Fig. S2.

The liposomes were obtained with a solution of 1-palmitoyl-2-oleyl-sn-glycero-3-phosphocholine (POPC), which spontaneously self-assembles into spherically closed bilayer membranes, where the polar heads are hydrated with solvent, whereas the hydrophobic hydrocarbon-chains of the phospholipid molecules interact with each other (conditions: 114 mg POPC in 1.5 ml 10 mM phosphate buffer, pH 7.4, 140 mM NaCl) (Fig. 2A)^21^,^22^,^23^. Emerging studies indicate that the more homogeneous the nanoparticles are, the better their performance^31^,^32^. So, to obtain liposomes with a homogeneous diameter, the solution was extruded 10 times through a double polycarbonate filter with pore size of 50 nm using compressed N_2_ (20–40 bar)^21^,^22^,^23^. The sizes of the liposomes were estimated by nanoparticle tracking analysis (NTA). The liposomes for imaging experiments have been marked with cyanine dye (Cy5), which was encapsulated in the liposomes (Fig. S2). The plots of Fig. 2B show that POPC liposomes have slightly different sizes depending whether they contain or not Cy5: diameter = 70 nm with Cy5; diameter = 85 nm without Cy5. The average amount of incorporated dye was ~0.5 mol % Cy5 per liposome.

After loading the liposomes with the G4-decoy and the cell penetrating peptide (TAT or R8), the number of effector molecules *per* liposome were: 96·ODN and 752·CPP *per* Cy5-liposome (70 nm); 142·ODN and 1137·CPP *per* liposome without Cy5 (85 nm) (see Experimental section).

**Figure 3:**
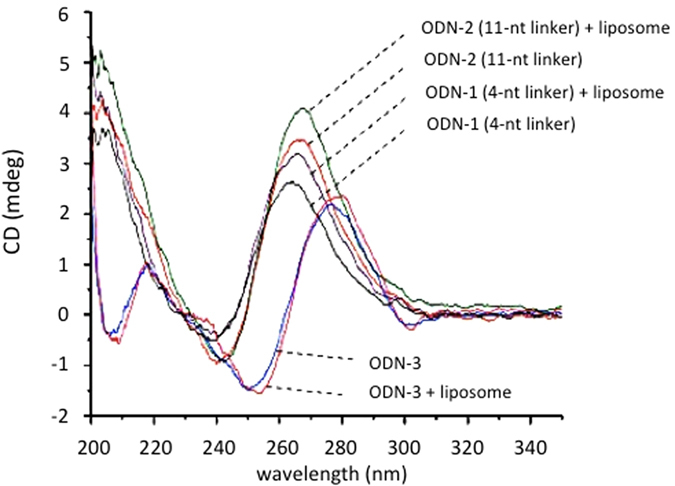
Circular dichroism spectra in phosphate buffer of G4-decoy ODN-1 and ODN-2 as free molecules (conc. 2.0 μM) or anchored to POPC liposomes (142 ODN/liposome). ODN-3 is a non G4 oligonucleotide used as a control.

Next, we interrogated if the G4-decoy **2998** maintains its folded structure also when it is anchored to the liposome surface through its two palmityl chains inserted close to the oligonucleotide 3′ end. To address this question we performed CD experiments which showed that ODN-3, free or attached to the liposomes, gave the same spectrum with a maximum at 278 nm and a minimum at 250 nm, which is indicative of an unstructured oligonucleotide, in keeping with the fact that it lacks a G4-motif (Fig. 3). By contrast, ODN-1 and ODN-2, that fold into a G4-DNA structure, exhibit an enhanced ellipticity at 260 nm typical of a parallel or mixed paerallel/antiparallel G-quadruplex^33^. Note that **2998** anchored to the liposome surface shows a 260 nm-ellipticity slightly stronger than that exhibited by the liposome free G4-decoy. This suggests that the oligonucleotide assumes its typical folded structure more efficiently when the alkyl chains are bound to and thus sequestered by the lipid bilayer of the liposomes.

**Figure 4:**
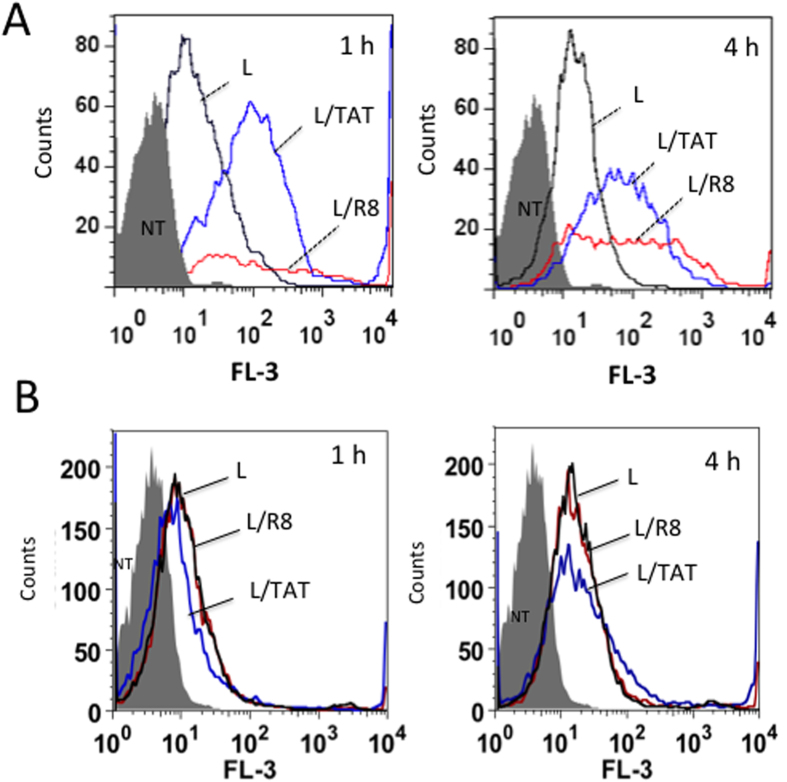
(**A**) FACS analyses of Panc-1 (**A**) and BxPC-3 (**B**) pancreatic cancer cells treated for 1 and 4 h with 360 pmol ODN-1 anchored to liposomes (96·ODN *per* liposome) loaded with Cy5 (L), Cy5/TAT (L/TAT) or Cy5/R8 (L/R8) (TAT and R8 are membrane anchored to the outer surface of the liposomes).

The bioactivity of the designed liposomes has been analysed mainly in Panc-1 cells, in which *KRAS* carries one of the most common mutation detected in patients affected by pancreatic adenocarcinoma (G12D, 12Gly→Asp)^34^. In order to evaluate their cell-penetrating capacity, POPC liposomes were marked with cyanine 5 (Cy5), which emits an intense fluorescence peak at 660 nm upon excitation at 540 or 620 nm. Cy5 was encapsulated in the liposomes by mixing the dye with POPC followed by extrusion and removal of excess dye by dialysis. Figure 4A shows a typical FACS analysis of Panc-1 cells treated for 1 and 4 h with Cy5-marked liposomes functionalized with ODN-1 (96·ODN-1 *per* liposome) (L) and CPP [752·(TAT or R8) *per* liposome] (L/TAT or L/R8). It can be seen that without CPP, the liposomes with ODN-1 were poorly taken up by the cells, as indicated by a modest shift to the right of the fluorescence peak (from FL-3~ 3 to FL-3~ 14). In contrast, when the liposomes were functionalized with both ODN-1 and CPP, their capacity to enter into the cells significantly increased. After 1 h of incubation, TAT-liposomes showed a fluorescence peak at FL-3~ 200, while at 4 h the peak occurred at FL-3~ 170, indicating that after an initial uptake the intracellular concentration of TAT-liposomes slightly decreased (probably due to some dissociation of the encapsulated Cy5). R8-liposomes entered into Panc-1 cells in a more complex way. While the cells treated with TAT-liposomes form a uniform population typified by a Gaussian peak of fluorescence, suggesting the presence of only one mechanism of transport, R8-liposomes instead give rise to a more heterogeneous cell population, as the fluorescence of the treated cells showed a broader peak after 1 and 4 h of incubation, which reflects more than one entry pathways: may be receptor-mediated endocytosis and direct fusion with cell membrane. Panel B shows the liposome uptake in pancreatic BxPC-3 cancer cells, which contrarily to Panc-1 are not *KRAS* mutated. The uptake in these cells is less efficient and does not seem to increase with CPP, suggesting a significant heterogeneity of pancreatic cancer cells. FACS analysis was performed also with MIA PaCa-2 cells, which bear a point mutation in exon 1, G12C (12Gly→Cys). The results are reported in Fig. S3, showing that POPC liposomes enter efficiently in this cancer cell line. Together, our data indicate that the liposomes penetrate more efficiently into *KRAS*-mutated cell lines.

**Figure 5:**
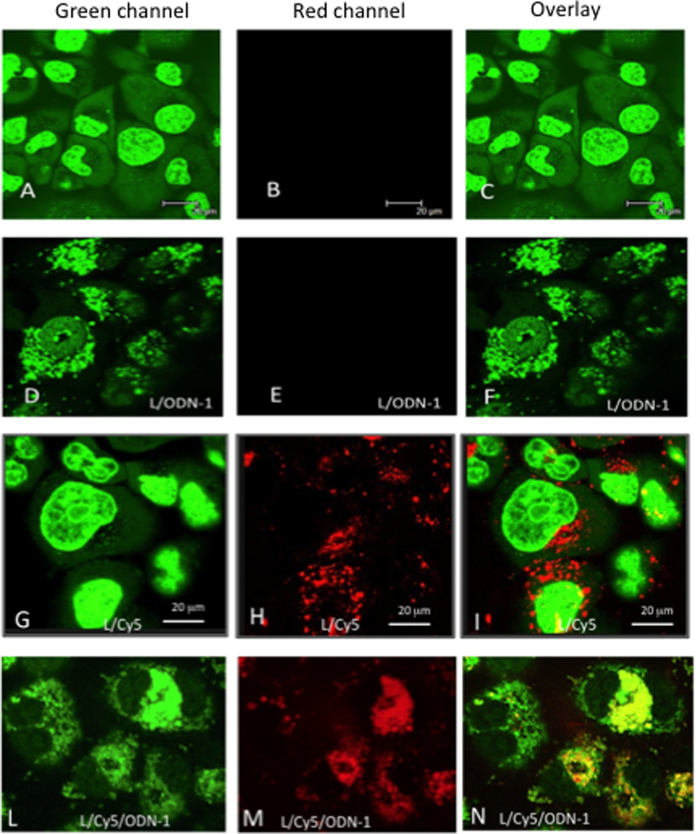
(**A–C**) Confocal microscopy images of viable Panc-1 cells treated with Syto-16: (**A**) green channel, (**B**) red channel, (**C**) overlay image; (**D–F**) Panc-1 cells treated with liposomes loaded with TAT/ODN-1 for 24 h and with Syto-16 for 2 h; (**G–I**) Panc-1 cells treated with liposomes loaded with Cy5/TAT for 24 h and with Syto-16 for 2 h; (**L–N**) Panc-1 cells treated with liposomes loaded with Cy5/TAT/ODN-1 for 24 h and with Syto-16 for 2 h. Concentration of ODN-1 in the experiments: 142·ODN *per* liposome without Cy5; 96·ODN *per* liposome with Cy5.

**Figure 6:**
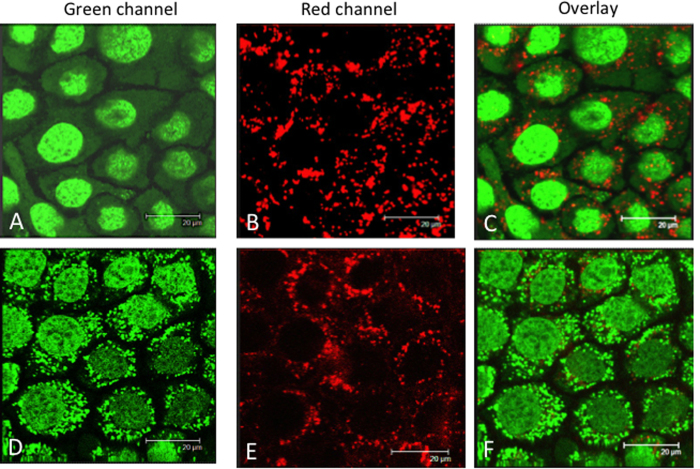
(**A–C**) Confocal microscopy images of viable BxPC-3 cells treated with liposomes loaded with Cy5/TAT for 24 h and with Syto-16 for 2 h: (**A**) green channel, (**B**) red channel, (**C**) overlay image; (**D–F**) BxPC-3 cells treated with liposomes loaded with Cy5/TAT/ODN-1 for 24 h and with Syto-16 for 2 h. Concentration ODN-1 in the experiments is 96·ODN *per* liposome.

Next, we examined the liposome uptake by confocal microscopy (Fig. 5). Panels A-C show Panc-1 viable cells stained with SYTO-16, a vital dye increasing its green fluorescence quantum yield upon binding to DNA and RNA^35^. SYTO-16 incubated for 2 h with the cells stains the nucleus in green much more than the cytoplasm, whose fluorescence appears slightly punctuated due to the binding of the dye to mitochondrial DNA. When Panc-1 cells were treated for 24 h with liposomes functionalized only with TAT and ODN-1 and for the last 2 h of incubation with SYTO-16 as well, a strongly punctuated fluorescence was visible in the cytoplasm, mainly in the perinuclear region (Panels D–F). This is due to the binding of SYTO-16 to the DNA anchored to the liposomes. Note that the nucleus appears now less green than in panel A because most SYTO-16 binds to the G4-decoy ODN-1 anchored on the liposomes surface (142·ODN-1 *per* liposome). Expectedly, the red channel did not give any signals as the liposomes were not marked with Cy5. Panels G-I show Panc-1 cells treated for 24 h with liposomes functionalized with TAT, Cy5 and with SYTO-16 for 2 h. As in panel A, the nuclei look more stained in green than the cytoplasm, where in this case the punctuated fluorescence is more visible. As expected, the red channel shows the distribution of the liposomes marked with Cy5. The liposomes are localized in the cytoplasm. As the red spots do not co-localize with the faint green spots, we can conclude that the liposomes do not internalize in the mitochondria. In panels L-N, we show Panc-1 cells treated with liposomes functionalized with TAT, Cy5 and ODN-1 (96·ODN-1 *per* liposome) for 24 h and with SYTO-16 for the last 2 h of incubation. As observed in panel D, most SYTO-16 is bound to ODN-1 anchored to the liposomes, so the nucleus appears poorly stained, while the cytoplasm where the liposomes accumulate appears strongly stained in green. As the liposomes are now also marked with Cy5, the red channel shows a fluorescence that co-localize with the green fluorescence of the cytoplasm. A close inspection of the micrographs shows that a small fraction of liposomes functionalized with ODN-1 is also localized in the nucleus. The confocal analyses performed with the various formulations of liposomes indicate that these nanoparticles accumulate primarily in the cytoplasm of pancreatic cancer cells. The above confocal experiments were carried out with viable cells. We also analysed the uptake in viable BxPC-3 pancreatic cancer cells (Fig. 6). Panels A, B and C show the cells treated with liposomes loaded with TAT and Cy5 for 24 h and with SYTO-16 for the last 2 h of the incubation. As observed with Panc-1 cells, the liposomes stained in red appear clearly localized in the cytoplasm (only a small fraction of liposomes is marked with Cy5). Panels D, E and F show the cells treated with liposomes functionalized with TAT, Cy5 and ODN-1 (96 ODN-1 *per* liposome). As with Panc-1 cells, SYTO-16 binds to ODN-1 bound to the liposomes and thus the cytoplasm appears strongly punctuated in green. The confocal images of viable MIAPaCa-2 are shown in Fig. S4. In addition, we have also analysed the uptake of Panc-1 cells fixed on glass, we obtained results in keeping with the analysis conducted with the viable cells (Fig. S5).

**Figure 7:**
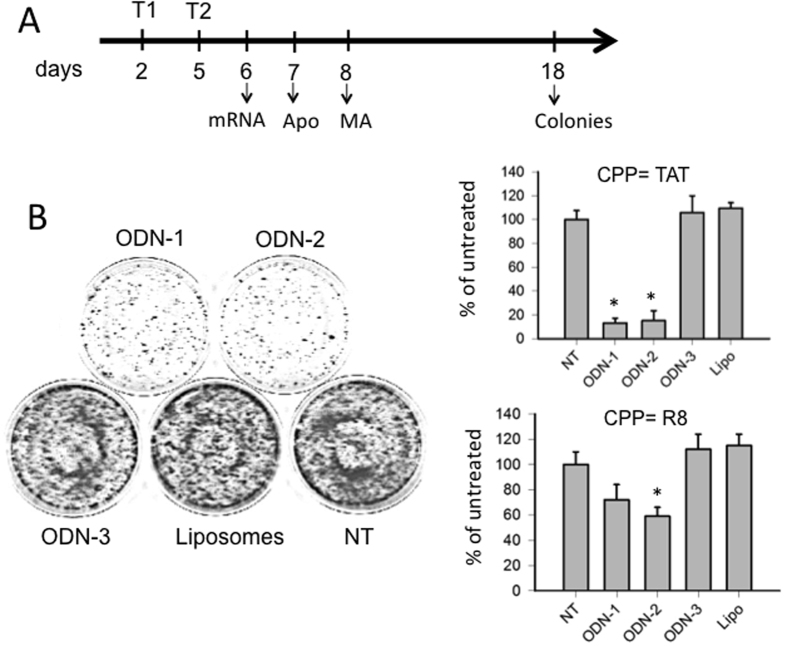
(**A**) Schematic representation on a temporal scale of the experiments performed with the POPC liposomes on Panc-1 cells. T1 and T2 indicate the liposome treatments; (**B**, left) Colony formation by Panc-1 cells untreated (NT) or treated with liposomes loaded with TAT only (Liposomes), or liposomes loaded with TAT and ODN-1 (ODN-1), TAT and ODN-2 (ODN-2) or TAT and ODN-3 (ODN-3) [each dish was treated with 72 pmol ODN anchored to POPC liposome (142·ODN *per* liposome]; (**B**, right) Histograms showing the percentage of colonies in untreated or liposome-treated cells, liposomes loaded with TAT (top panel), liposomes loaded with R8 (bottom panel). Peptide loading: 1137·peptides *per* liposome. NT = untreated cells, ODN 1-3, cells treated with liposomes loaded with ODN and CPP (TAT or R8), Lipo = cells treated with unloaded liposomes. *P < 0.05.

**Figure 8:**
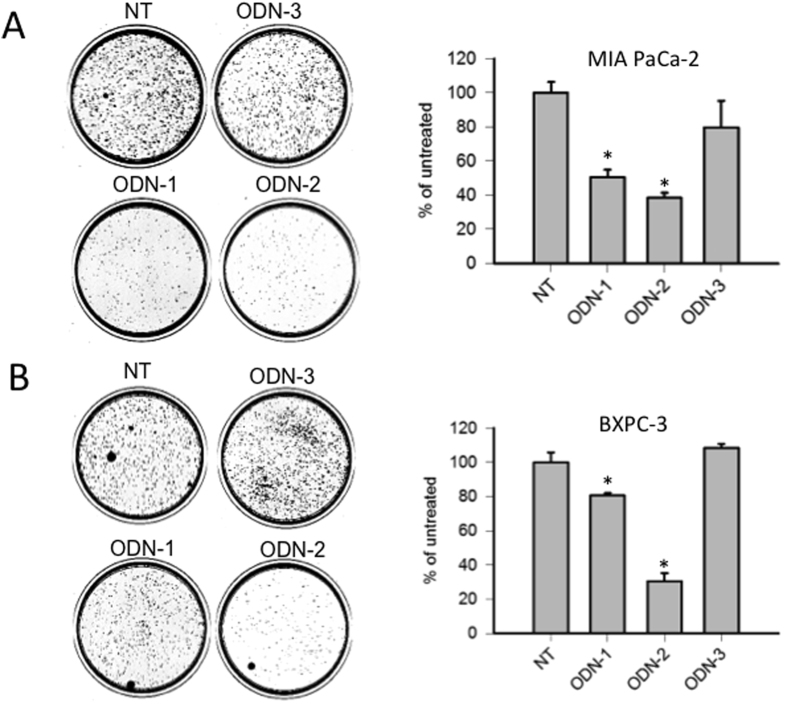
(**A**) Colony formation by pancreatic adenocarcinoma MIA PaCa-2 cells untreated (NT) or treated with liposomes loaded with TAT and ODN-1 or ODN-2 or ODN-3 [each dish was treated with 72 pmol ODN anchored to POPC liposome (142·ODN *per* liposome]. The histograms show the percentage of colonies with respect to untreated cells (NT) or cells treated with TAT liposome loaded with ODN-1 or ODN-2 or ODN-3. Peptide loading: 1137·peptides *per* liposome; (**B**) as in A but with pancreatic adenocarcinoma BxPC-3 cells. *P < 0.05.

As G4-decoy **2998** binds to proteins recognizing G4-proximal (PARP-1, Ku70, hnRNP A1, MAZ)^9^, it inhibits *KRAS* transcription by a decoy mechanism. As a consequence, the proliferation and clonogenic potential of Panc-1 cells are reduced by the G4-decoy oligonucleotide^12^. To investigate the clonogenic effects of lipid-modified **2998** anchored to POPC liposomes, we first treated Panc-1 cells (bearing the point mutation G12D in exon 1^34^) with the liposomes functionalized with TAT and ODN-1 (142 ODN-1/liposome). We started the experiment with one single treatment. After 13 days of incubation, we observed that the G4-decoy did not produce the expected inhibition of colony formation (not shown). We therefore performed two treatments, at day 2 and 5, as previous experiments showed that in this way **2998** exhibited activity in Panc-1 cells^12^. The experiment outlined is shown in Fig. 7A. The cells were treated twice with TAT- or R8-liposomes, loaded with ODN-1, ODN-2 or ODN-3. 13 days after the second treatment, the colonies formed were stained and counted. The results reported in the histogram showed that TAT-liposomes loaded with G4-decoy ODN-1 or ODN-2 strongly reduced colony formation to ~10% of the control (Panc-1 cells untreated or treated with ODN-3) (Fig. 7B). R8-liposomes reduced colony formation to only 60% of the control: a finding that may reflect the complexity of their uptake suggested by the FACS data (Fig. S6). To know if the observed strong inhibition of colony formation mediated by the G4 decoys is cell line dependent, we extended our analysis to other two adenocarcinoma pancreatic cancer cells: MIA PaCa-2 carrying the G12C (12Gly→Cys) mutation in exon 1/codon 12 and BxPC-3 carrying wild type codon 12/exon 1^34^. The results reported in Fig. 8 show that the G4-decoys ODN-1 and ODN-2, but not the control ODN-3, significantly decrease the clonogenic potentials also in these cells, both in terms of number and size of colonies. This demonstrates that the G4-decoys anchored to the liposomes did not lose the anticlonogenic activity that was observed with the free G4-decoys delivered with jet-PEI^11^.

**Figure 9:**
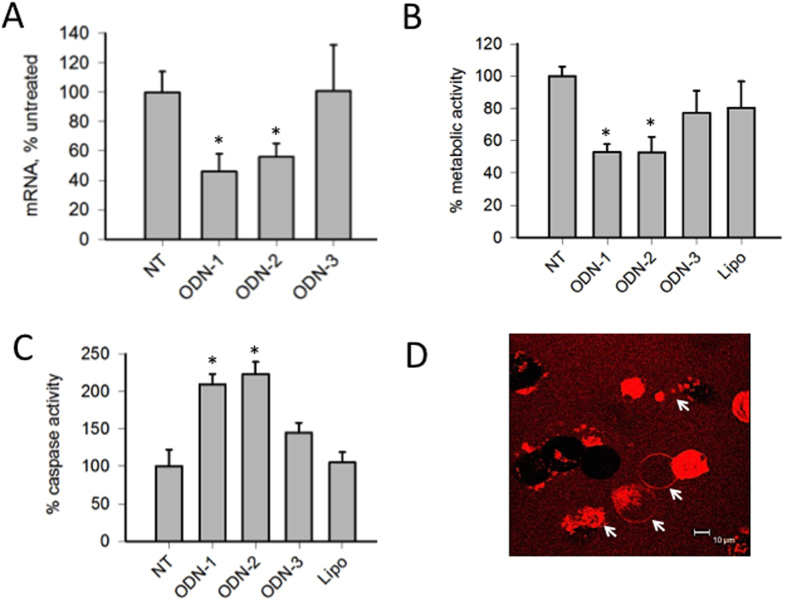
(**A**) Level of *KRAS* mRNA in Panc-1 cells untreated (NT) or treated with liposomes loaded with TAT and ODN-(1/2/3), (142·ODN *per* liposome)*P < 0.05; (**B**) Metabolic activity of Panc-1 cells untreated or treated with liposomes loaded with TAT and ODN-(1/2/3), (142·ODN *per* liposome). Lipo indicates cells treated with TAT-liposome unloaded with ODN. The ordinate report the % of the control (untreated cells, NT), *P < 0.05; (**C**) Activation of caspases 3/7 in Panc-1 cells untreated or treated with liposomes loaded with TAT and ODN-(1/2/3), (142·ODN *per* liposome). The ordinate represent the % of the control, *P < 0.05; (**D**) Panc-1 cells treated with TAT liposomes loaded with Cy5 and ODN-1. The images report the red fluorescence emitted by Cy5. The arrows show the typical bubbling of apoptotic cells and cells fragments produced by apoptosis (apoptotic bodies).

As a next step, we determined, by quantitative RT-PCR, the effect of TAT-liposomes loaded with ODN-1, ODN-2 and ODN-3 on *KRAS* mRNA (Fig. 9A). We observed that the G4-decoy oligonucleotides ODN-1 and ODN-2 reduced the level of mRNA to about 50% of the control (mRNA level in untreated or ODN-3 treated cells), suggesting that the different length of the spacers between the G4 sequence and the lipid chains does not affect the decoy activity. Oligonucleotide ODN-3, which is unable to fold into a G-quadruplex, showed no inhibitory effects on *KRAS* transcription. The results are in agreement with a previous study carried out with **2998** delivered to the cells with PEI^12^. As *KRAS* is an oncogene that reprograms the metabolism of cancer cells^6^,^7^, we examined the metabolic activity in Panc-1 cells untreated or treated with the liposomes. The metabolic activity of Panc-1 cells was measured with resazurin: viable cells with an active metabolism reduce resazurin into resorufin, which is a pink and fluorescent molecule^36^. It can be seen that ODN-1 and ODN-2 reduce the metabolic activity to ~50% of the control (untreated or ODN-3-treated cells) (Fig. 9B). As we previously demonstrated, the G4-decoy oligonucleotide **2998** reduces both cell growth and clonogenic potential by activating apoptosis. We therefore investigated if this mechanism is also activated when **2998** is delivered with TAT-liposomes. We measured the activation of caspases 3/7 and found indeed that ODN-1 and ODN-2, but not ODN-3, activate the caspases in Panc-1 cells (Fig. 9C). This was also observed by confocal microscopy analyzing living cells treated with TAT-liposomes loaded with Cy5/ODN-1. It can be seen that some cells are completely red due to a bulky internalization of liposomes. Note that some completely red cells show the typical signs of apoptosis (indicated by arrows): bubbling and shrinkage into cell fragments (Fig. 9D).

In conclusion, in this article we have demonstrated that a G4-decoy oligonucleotide with anti-*KRAS* activity can be delivered with POPC liposomes marked with TAT or R8 peptide. Both functionalities, G4-decoy and TAT peptide, bear two palmityl chains that allow their anchoring to the liposome surface. As the lipid-modified G4-decoys anchored to the liposomes display their bioactivity in pancreatic cancer cells, POPC liposomes may be suitable carriers for *in vivo* delivery of therapeutic oligonucleotides including the G4-decoys as well as antisense and miRNAs. Work is in progress in our laboratories along this direction.

## Experimental section

### Synthesis of modified oligonucleotides

Oligonucleotides were synthesized on an ExpediteTM 8900 nucleic acid synthesis system (Perceptive Biosystems Inc.). The syntheses were performed on a 1.0 μmol scale on GE Healthcare Custom Primer Support^TM^ T40s using standard conditions for automated synthesis with DCI as activator. However, the lipid modified phosphor amidite was dissolved in 2:1 DCE:MeCN at a concentration of 0.1 M, 42^®^ was used as activator (Proligo reagent/Sigma-Aldrich) and the coupling time was increased to 20 min. The coupling time for TINA was 5.2 min. The DMT protecting group on the last nucleotide in the sequence was removed. After deprotection and cleavage from the solid support using standard conditions (conc. NH_3_(aq.) over night at 55 °C), the oligonucleotides were purified by HPLC. Purification was performed using a DIONEX Ultimate 3000 with a DIONEX Acclaim^®^ C18 3 μm 300 Å reversed phase column with UV detection at 260 nm. A flow rate of 1 mL/min. and a column temperature of 50 °C using the following gradient program: 2 min. isocratic with 0.05 M triethyl ammonium acetate (TEAA) followed by a 8 min. linear gradient to 40% 1:3 H_2_O:MeCN and a 20 min. linear gradient to 100% 1:3 H_2_O:MeCN which was continued isocratic for 10 min., was used. After HPLC purification, the oligonucleotides were desalted using a NAP 10 column (Sephadex G25 grade). Mass spectra of oligonucleotides were recorded using a Bruker Daltonics microflex LT MALDI-TOF.

### Synthesis of lipid-modified peptides

The peptides were synthesized on a Liberty 1 Microwave Peptide Synthesizer using a Rink Amide resin, Fmoc as protecting group, PyBOP as activator, DIPEA as base and an amino acid concentration of 0.2 M for unmodified amino acids and 0.1 M for the lipid-modified amino acid. R was coupled twice. After synthesis, the peptide was cleaved from the resin and deprotected by treatment with 92.5:2.5:5 TFA:TIS:DMB under microwave conditions at 60 °C for 1800 s. After filtration and lyophilization, the peptide was dissolved in 99:1 H_2_O:TFA and purified by HPLC using a DIONEX Ultimate 3000 with a Waters XBridge^TM^ Prep C18 5 μm 10 × 150 mm column. Purification was performed with UV detection at 214 nm, a column temperature of 36 °C, a flow rate of 1 mL/min. and the following gradient program: 5 min. isocratic with 5:95A (90:10:0.1 MeCN:H_2_O:TFA):B (100:0.1 H_2_O:TFA), followed by a 15 min. linear gradient to 10:90A:B and a 50 min. linear gradient to 95:5A:B, which was continued isocratic for 3 min. before a 4 min. linear gradient to 5:95A:B, which was continued for18 min.

### Preparation of POPC liposomes

Liposomes were extruded using a LIPIX Extruder, Northern Lipids. 114 mg 1-palmitoyl-2-oleyl-*sn*-glycero-3-phosphocholine (POPC) was suspended in 1.5 mL 10 mM phosphate buffer (10 mM NaH_2_PO_4_. 2 H_2_O, 5 mM Na_2_HPO_4_, 140 mM Na^+^, pH 7.4) (100 mM POPC) and the resulting solution was extruded 10 times through two stacked polycarbonate filters with a pore size of 50 nm using compressed N_2_ (~30 bar).

### Preparation of Cy5-labelled POPC liposomes

1.5 mg sulfo-Cyanine 5 NHS ester was dissolved in 1.0 mL 10 mM phosphate buffer (10 mM NaH_2_PO_4_. 2 H_2_O, 5 mM Na_2_HPO_4_, 140 mM Na^+^, pH 7.4), and 76 mg 1-palmitoyl-2-oleyl-*sn*-glycero-3-phosphocholine (POPC) (corresponding to a concentration of 100 mM POPC and 0.5 mol% Cy5 after dialysis) was suspended herein. The resulting solution was extruded 20 times through two stacked polycarbonate filters with a pore size of 50 nm using compressed N_2_ (~30 bar). Dye not encapsulated in the liposomes was removed by dialysis against 3 changes of buffer.

### Nanoparticle Tracking Analysis

The sizes of the liposomes were determined using Nanoparticle Tracking Analysis (NTA). NTA measurements were performed with a NanoSight LM10-HS equipped with an Andor Lucas EMCCD camera, a LM14 temperature controller and a laser diode operated at 404 nm. The data was analysed using the NanoSight NTA 2.1 software.

Due to the relatively low particle concentration necessary for NTA (10^8^-10^9^ particles/mL), the samples with dye-labelled liposomes were diluted 250000-fold and the unlabelled liposomes 10000- to 100000-fold with phosphate buffer (10 mM NaH_2_PO_4_. 2 H_2_O, 5 mM Na_2_HPO_4_, 140 mM Na^+^, pH 7.4). All measurements were carried out in duplicate at 23 °C.

### Circular dichroism spectra

CD spectra were obtained using the same samples which was used for *T*_m_-measurements and were collected at 37 °C using a JASCO J-815 CD Spectrometer and cuvette with a 2.00 mm path length. The spectra have been collected in phosphate buffer and the oligonucleotide concentration was 2.0 μM for both free oligonucleotides and oligonucleotides anchored to POPC liposomes (142 ODN/liposome). The measurements were recorded from 200 to 350 nm at a scanning speed of 50 nm/min, a data pitch of 0.2 nm and a response time of 4 s. The final spectra are the average of three measurements.

### Cell culture and transfection

The cells used in this study are: (i) human pancreatic adenocarcinoma cells (Panc-1) with a *KRAS* G12D (12 Gly→Asp) mutation; (ii) human pancreatic adenocarcinoma cells (MIA PaCa-2) with a *KRAS* G12C (12 Gly→Cys) mutation; (iii) human pancreatic adenocarcinoma cells (BxPC-3) with wild-type *KRAS*. All cells bear mutant TP53^29^. Human pancreatic cancer cells (Panc-1) were maintained in exponential growth in Dulbecco’s modified Eagle’s medium (DMEM) containing 100 U/ml of penicillin, 100 mg/ml of streptomycin, 20 mM L-glutamine and 10% fetal bovine serum (Euroclone, Milano, Italy). The cell lines have been genotyped by Microsynth (CH) to verify their identity. As expected, they matched 100 % to the DNA-profiles of the cell line of PANC-1 (ATCC^®^ CRL-1469TM), MIA PaCa-2 (ATCC^®^ CRM-CRL-1420TM), BxPC-3 (ATCC^®^ CRL-1687TM).

POPC (16 mM) was mixed with 4 nmol oligonucleotide in 100 μl phosphate buffer (10 mM NaH_2_PO_4_, 5 mM Na_2_HPO_4_, 140 mM NaCl pH 7.4). After overnight incubation at room temperature, 90.8 μg of peptide were added to the solution and let to incubate for 30 minutes (liposome mix). In order to achieve membrane anchoring of the intended number of copies of oligonucleotides (ODNs) and respectively peptides (CPPs) per liposome we calculated the surface area of the liposomes based on their measured size and the number of liposomes in solution based on their measured size and the lipid (POPC) concentration used. 2 μl of each ODN stock solutions corresponded to 1 nmol of the respective ODN and 1 μl of each peptide stock solution corresponded to the intended copy number of peptides per liposome. For all liposome formulations a statistic distribution of ODNs and CPPs during membrane anchoring to the surface of the liposomes was assumed.

For confocal images, 4.5 μl of liposome mix were added to 1 μl cell medium and the final solution used to treat the cells. For the other experiments (FACS, clonogenic assays, apoptopsis, RT-PCR and metabolic activity) the liposome mix was obtained by adding 0.36 nmol oligonucleotide in 100 μl buffer (POPC and peptide were scaled down accordingly). Then 100 μl of liposome mix were in this case added to 0.5 μl cell medium and the final solution used to treat the cells. After 48 h the treated cells of each well were divided in 3 parts and seeded in 3 wells. After 24 h a second transfection following the same protocol was performed. This protocol was used for the qRT-PCT, metabolic activity, clonogenic and apoptosis assays.

### RNA Extraction and Real-time PCR

Panc-1 cells (10^4^) were transfected in a 96-well plate with 72 pmol oligonucleotide, as described above. RNA was extracted by using iScript™ RT-qPCR Sample Preparation Reagent (Bio Rad) following the manufacturer’s instructions 24 h after the second transfection. For cDNA synthesis 1.25 μl of RNA (extracted from about 10^3^cells) was heated at 55 °C and placed in ice. The solution was added to 11.25 μl of mix containing (final concentrations) 1x buffer, 0.01 M DTT (Invitrogen), 1.6 μM primer dT (MWG Biotech, Ebersberg, Germany; d(T)16), 1.6 μM Random hexamer primers (Microsynth), 0.4 mM dNTPs solution containing equimolar amounts of dATP, dCTP, dGTP, and dTTP (Euroclone, Pavia, Italy), 0.8 units/μl RNase OUT, and 8 units/μl of Maloney murine leukemia virus reverse transcriptase (Invitrogen). The reactions were incubated for 1 h at 37 °C and stopped with heating at 95 °C for 5 min.

Real-time PCR multiplex reactions were performed with 1 x Kapa Probe fast qPCR kit (KAPA Biosystems, Wilmington, MA, USA) for *KRAS* and housekeeping genes β2-microglobulin and HPRT, 1.0 μl of cDNA in 10 μl final and primers/probes at the following concentrations: for *KRAS*, the probe was FAM-TACTCCTCTTGACCTGCTGTG-BHQ1 (accession No. NM_033360, from 352 to 372, 90 nM), the sense primer was 5′-C GAATATGATCCAACAATAGAG (from 271 to 292, 180 nM) and the antisense primer was 5′-ATGTACT GGTCCCTCATT (from 379 to 396, 180 nM). For β2- microglobulin accession n. NM_004048 probe ROX-TATGCCTGCCGTGTGAACC-BHQ2 (from 352 to 370, 60 nM), the primer sense was 5′-CCCCACTGAAAAAGATGA (from 333 to 350, 100 nM), the primer antisense was 5′-CCATGATGCTGCTTACAT (from 415 to 432, 100 nM). For HPRT accession n. NM_000194 probe 5′-Cy5-CTTGCGACCTTGACCATCTT-BHQ2 (from 633 to 652, 180 nM), the primer sense was 5′-CTTGATTGTGGAAGATATAATTG (from 557 to 575, 210 nM), the primer antisense was 5′-TATATCCAACACTTCGTGG (from 672 to 690, 230 nM). The PCR cycle was: 3 min at 95 °C, 50 cycles 10 s at 95 °C, 60s at 58 °C. PCR reactions were carried out with a CFX-96 real-time PCR apparatus controlled by an Optical System software (version 3.1) (BioRad Laboratories, CA, USA). *KRAS* mRNA was normalized with the two housekeeping genes.

### Metabolic activity and clonogenic assays

The metabolic activity (MA) of Panc-1 cells was measured on a 96-well plate where each well, containing 10^4^ cells, was transfected twice with the POPC liposomes loaded with ODN (142 ODN/liposome). The MA was measured 72 h after the second transfection by a resazurin assay: 25 μM resazurin was added to the cell medium, and the fluorescence was measured after 1 h (Ex 535 nm; Em 590 nm) with a spectrofluorometer EnSpire 2300 Multilabel Reader (Perkin Elmer).

For colony-forming assay, Panc-1, BxPC-3 and MIAPaCa-2 cells were transfected with POPC liposomes loaded with ODN (142 ODN/liposome). 18 h following the second transfection, the wells were treated with trypsin and one third of the volume of each well was seeded on 60-mm diameter plate and the cells were let to grow under normal culture conditions. After 13 days, the cells were stained with 2.5% methylene blue in 50% ethanol for 10 min. Colonies of >50 cells were counted with Image Quant TL software (Image Scanner, Amersham).

### Apoptosis assays

Caspase assay was performed with Apo-ONE^TM^ Homogeneous Caspase-3/7 Assay (Promega), according to the manufacturer’s protocol. The assay was performed 48 h after the second liposome treatment.

### Cytofluorimetric analysis

FACS analyses were carried out on Panc-1 cells (2.5 × 10^4^) treated with 360 pmol oligonucleotide ODN-1 anchored to liposomes (96 ODN-1/liposome) for 1, 2 and 4 h. The cells were harvested at various time points after liposome transfection, washed with PBS and immediately analysed by FACSAria III flowcytometer (Becton-Dickinson, San Jose, CA, USA). A minimum of 10^4^ cells for each sample were analyzed. The signal was detected by FL3 (680 nm) channel in log scale. The uptake data was obtained with FLOWJO flow cytometry analysis software (FLOWJO LLC, Ashland OR USA).

### Confocal imaging

Confocal microscopy experiments with unfixed viable cells (Panc-1, BxPC-3 and MIAPaCa-2) have been performed by plating the cells on a glass bottom plate (about 3 × 10^5^ cells). After 24 h the cells were treated for 24 h with various liposome formulations and for the last 2 h with 100 nM SYTO 16, which stains the nucleus. As liposome formulations we used: (i) POPC liposomes (72 nmol POPC) loaded with 180 pmol ODN-1 (142 ODN-1 *per* liposome) and TAT (1.4 nmol); (ii) POPC liposomes (72 nmol POPC) loaded with TAT (1.4 nmol) and Cy5 (1.4 nmol); (iii) POPC liposomes (72 nmol POPC) loaded with 180 pmol ODN-1 (96 ODN-1 *per* liposome) and TAT (1.4 nmol) and Cy5 (1.4 nmol).

For confocal experiments with glass-fixed Panc-cells, we plated (3 × 10^5^) on a coverslip placed in a Petri dish (diameter 35 mm) and after 24 h treated with liposomes (36 nmol POPC) loaded with 0.8 nmol ODN-1 (380 ODN-1 *per* liposome), TAT (0.7 nmol) and Cy5 (0.7 nmol) for 4 h. The cells were treated also with SYTO 14 for 1 h. The cells have been washed twice with PBS and fixed with 3% paraformaldehyde (PFA) in PBS for 20 min. After washing with 0.1 M glycine, containing 0.02% sodium azide in PBS to remove PFA and Triton X-100 (0.1% in PBS), the nuclei have been stained for 5 min with Hoechst (3 ng/μL in PBS). The cells were analysed on a Leica TCS SP1 confocal imaging system.

## Additional Information

**How to cite this article**: Cogoi, S. *et al*. Lipid-modified G4-decoy oligonucleotide anchored to nanoparticles: delivery and bioactivity in pancreatic cancer cells. *Sci. Rep.* **6**, 38468; doi: 10.1038/srep38468 (2016).

**Publisher’s note:** Springer Nature remains neutral with regard to jurisdictional claims in published maps and institutional affiliations.

## Supplementary Material

Supporting Information

## Figures and Tables

**Table 1:** Modified G4-decoy oligonucleotides and CPP-modified peptides (TAT or R8).

ODN	Sequence	Mass of [M + H]^+^/g mol^−1^	
		Theoretical	Experimental
ODN-1	5′-GCG GTG TGG G**P**A AGA GGG AAG A**P**G GGG GAG GCA^L^ G^L^TT **Y** TTT	13338.7	13332.5
ODN-2	5′-GCG GTG TGG G**P**A AGA GGG AAG A**P**G GGG GAG GCA^L^ G^L^TT ATT ATT A**Y** TTT	15574.0	15574.6
ODN-3	5′-GCG TTG TCG C**P**A AGA CGC AAG ACG CGT AGT CAG TT**Y** TTT	12499.5	12524.5
TAT	EEE**XX**YGRKKRRQRRRE-NH_2_	2806.8	2807.7
R8	EEE**XX**RRRRRRRRE-NH_2_	2515.7	2517.8

P = *para*-TINA insertion; Y = DNA lipid modification; X = peptide lipid modification.
