# Supplementary material for: Lipid-modified G4-decoy oligonucleotide anchored to nanoparticles: delivery and bioactivity in pancreatic cancer cells
Source: Sci Rep. 2016 Dec 8;6:38468. doi: 10.1038/srep38468 (PMC5144097; doi:10.1038/srep38468)
Supplement: Supporting Information [file srep38468-s1.doc]

Supporting Information

Lipid-modified G4-decoy oligonucleotide anchored to nanoparticles: delivery and bioactivity in pancreatic cancer cells

**S. Cogoi,^a^ U. Jakobsen,^b,c^ E. B. Pedersen,^b^ S. Vogel,*^,b^ L. E. Xodo *^,a^**

*^a^ Department of Medical and Biological Sciences, P.le Kolbe 4, 33100 Udine, Italy.*

*^b^ Nucleic Acid Center, Institute of Physics and Chemistry, University of Southern Denmark, DK-5230 Odense M, Denmark.*

*^c^ PET & Cyclotron Unit, Department of Nuclear Medicine, Odense University Hospital, Sdr. Boulevard 29, 5000 Odense C, Denmark*

**(A)**

**
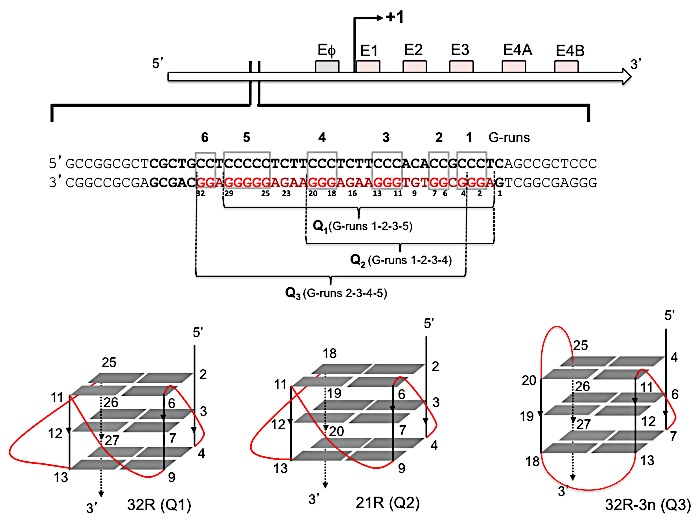
**

**(B)**


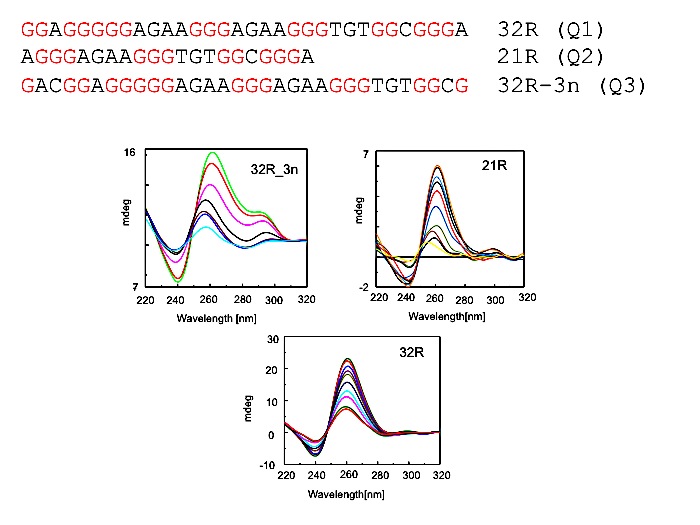


**Fig. S1**: (A) Primary structure of the G4-proximal sequence of the *KRAS* promoter upstream of TSS. DMS footprinting and CD experiments showed that sequence G4-proximal folds into a parallel 1/1/11 G-quadruplex with a kinked thymine in one strand, two 1-nt and one 11-nt loops (formed by G-runs 1-2-3-5, Q1, *T*_M_= 75 °C in 100 mM KCl). Further analyses showed that G4-proximal exhibits a remarkable structural polymorphism, as two truncated G4-proximal sequences composed by G-runs 1-2-3-4 and 2-3-4-5 fold also into a G-quadruplex, respectively with a parallel, *T*_M_= 56 °C, 100 mM KCl (Q2) and mixed parallel/antiparallel, *T*_M_= 50 °C, 100 mM KCl (Q3) topologies; (B) CD spectra as a function of temperature (from 20 to 90 °C) of the G-quadruplex motifs 32R, 21R and 32R-3n.


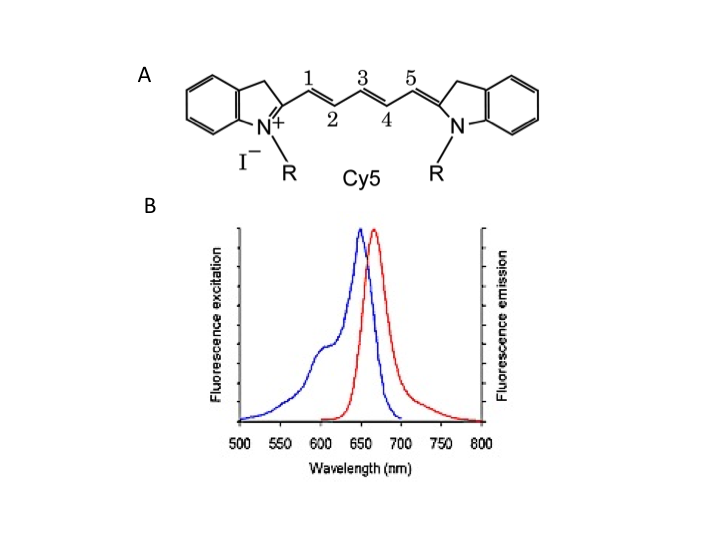


**Figure S2**: (A) Structure of cyanine dye Cy5 and (B) fluorescence excitation and emission spectra.

Ex: 650 nm; Em: 670 nm; quatum yield: 0.27 (Mujumdar B, et al. 1993, *Bioconjugate Chemistry* **4** (2): 105–111)


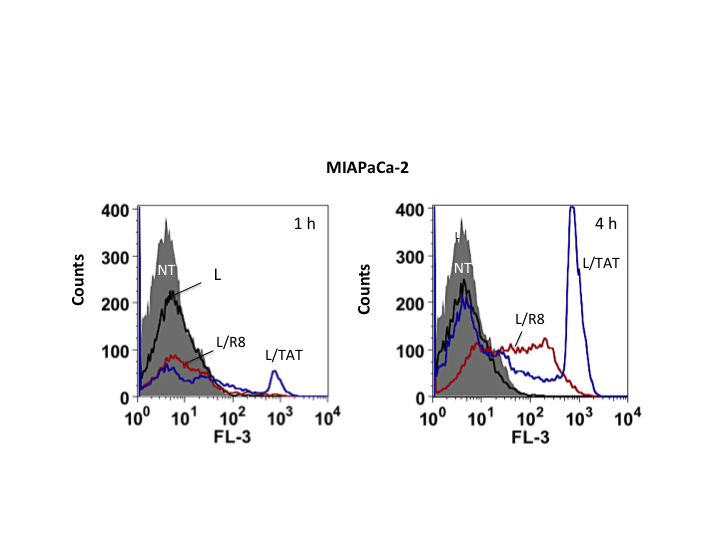


**Fig. S3** (A) FACS analyses of MIA PaCa-2 cells treated for 1 and 4 h with 360 pmol ODN-1 anchored to liposomes (96·ODN *per* liposome) loaded with Cy5 (L), Cy5/TAT (L/TAT) or Cy5/R8 (L/R8) (TAT and R8 are membrane anchored to the outer surface of the liposomes).

The graphs report only viable cells. Dead cells, debris and liposome aggregates have been “gated out”. Liposomes without CPP do not enter into the cells. Liposomes loaded with R8 show a broad uptake similar to that observed with Panc-1 cells. Instead, the uptake of liposome functionalized with the TAT peptide exhibit a heterogeneous uptake with cells that have taken up very little liposomes and cells with a high liposome load. This may be due to the fact that MIAPaCa-2 cells do not exhibit a homogeneous morphology: in culture there are single cells, loosely attached clusters and floating rounded cells. It is possible that one of these forms is more prone to internalize POPC liposomes.


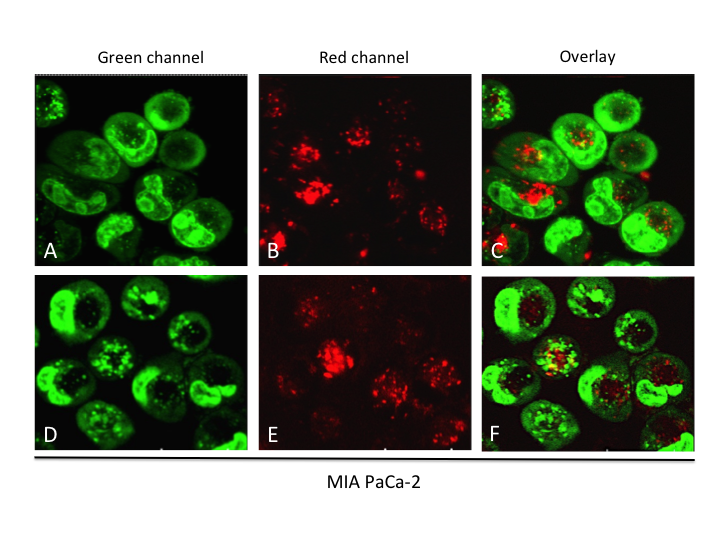


**Fig. S4**: (A-C) Confocal microscopy images of viable MIAPaCa-2 cells treated with liposomes loaded with Cy5/TAT for 24 h and with Syto-16 for 2 h: (A) green channel, (B) red channel, (C) overlay image; (D-F) Panc-1 cells treated with liposomes loaded with Cy5/TAT/ODN-1 for 24 h and with Syto-16 for 2 h. Concentration ODN-1 in the experiments is 96·ODN *per* liposome.


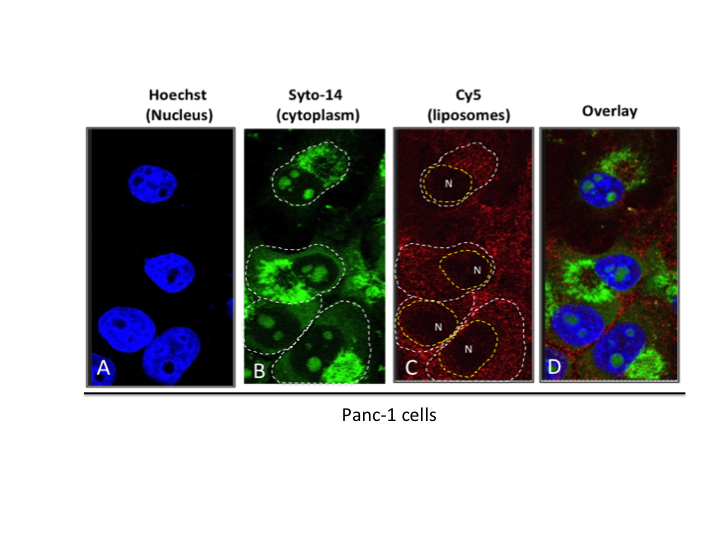


**Fig. S5.** Confocal microscopy images of Panc-1 cells treated with SYTO-14, Hoechst and liposomes loaded with ODN-1/TAT. Panc-1 cells have been fixed with paraformaldehyde. (A) Nuclei stained with Hoechst; (B) Cytoplasm stained with Syto-14; (C) Liposomes loaded with TAT, ODN-1 and Cy5. The contours of four cells are depicted in white, while the respective nuclei are outlined in yellow. Concentration ODN-1 in experiments: 0.8 nmol anchored to liposomes (380·ODN-1 *per* liposome); (D) overlay image.

The figure shows typical glass-fixed Panc-1 cells with the nucleus in blue (panel A) and the cytoplasm in green (panel B). Note that the cytoplasm is strongly stained in green due to the binding of the dye to RNA. As the cells were treated for 24 h with liposomes loaded with Cy5/TAT/ODN-1, the red channel shows the fluorescence emitted by Cy5 (panel C). The contour of the cells was given in order to facilitate the intracellular localization of the liposomes. The overlay of the three channels is shown in panel D. In agreement with the results obtained with living cells, the liposomes (red punctuated fluorescence) accumulate primarily in the cytoplasm of the cells, which shows a diffuse red fluorescence due to a homogeneous distribution of the liposomes. However, a close inspection of the images shows that a fraction of liposomes is also detected inside the nucleus.


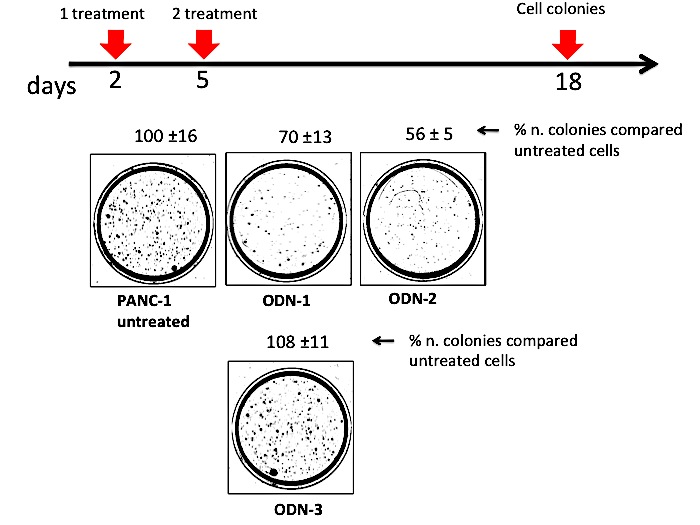


**Fig. S6**: Number of Panc-1 colonies 13 days after the second treatment with POPC liposomes loaded with the oligonucleotides indicated in the figures and with the peptide R8.

The numbers above the plates indicate the percentage of colonies compared to the colonies detected in untreated Panc-1 cells.
